# Distribution of Virulence and Pheromone-Associated Genes Among Colonizing *Enterococcus faecalis* Isolates Recovered Through Routine VRE Screening in Cluj-Napoca, Romania

**DOI:** 10.3390/life16081333

**Published:** 2026-08-14

**Authors:** Dan-Alexandru Toc, Matei-Stefan Dobrescu, Anca Livia Butiuc-Keul, Alexandru Botan, Irina-Maria Rusu, George Berar, Bogdan-Valentin Roznovan, Beata Dohi, Dan Vălean, Ioana Colosi, Carmen Costache

**Affiliations:** 1 Department of Microbiology, Iuliu Hatieganu University of Medicine and Pharmacy, 400012 Cluj-Napoca, Romania; toc.dan.alexandru@elearn.umfcluj.ro (D.-A.T.);; 2 Cluj County Emergency Hospital, 400000 Cluj-Napoca, Romania; 3 Department of Molecular Biology and Biotechnology, Faculty of Biology and Geology, Babeș-Bolyai University, 1 M. Kogalniceanu Street, 400084 Cluj-Napoca, Romania; 4 Centre for Systems Biology, Biodiversity and Bioresources, Babes-Bolyai University, 5–7 Clinicilor Street, 400006 Cluj-Napoca, Romania; 5 Discipline of Medical Informatics and Biostatistics, Faculty of Medicine, Iuliu Hațieganu University of Medicine and Pharmacy, 400349 Cluj-Napoca, Romania; 6 Faculty of Medicine, Iuliu Hatieganu University of Medicine and Pharmacy, 400012 Cluj-Napoca, Romania; 7 Department of Surgery, Iuliu Hațieganu University of Medicine and Pharmacy, 400012 Cluj-Napoca, Romania; 8 Regional Institute of Gastroenterology and Hepatology Octavian Fodor, 400162 Cluj-Napoca, Romania

**Keywords:** *Enterococcus faecalis*, vancomycin-resistant enterococci, virulence genes, colonization, quorum sensing, pheromone-responsive genes, antimicrobial resistance, hospital epidemiology

## Abstract

**Background**: *Enterococcus faecalis* is a common gastrointestinal commensal and an important opportunistic pathogen. While vancomycin resistance is well characterized, data on virulence-associated genes in colonizing isolates remain limited. **Methods**: A total of 44 colonizing *E. faecalis* isolates recovered from routine VRE screening (rectal swabs) were included in the study. The presence of the virulence-associated genes *asa*, *esp*, *eep*, *ccf*, and *cpd* was investigated by PCR. Antimicrobial susceptibility testing was performed using standardized methods, and isolates were subsequently classified as either vancomycin-resistant (*VRE*) or vancomycin-susceptible (*VSE*). A cumulative virulence gene score was calculated for each isolate and compared between the two groups. **Results**: Virulence-associated genes were heterogeneously distributed. Overall, the prevalence of the investigated virulence genes was *cpd* (33/44), *eep* (26/44), *esp* (24/44), *ccf* (22/44), and *asa* (8/44). Among these, only *esp* was significantly more prevalent in VRE than in VSE isolates (*p* < 0.01). VRE isolates showed a significantly higher median virulence gene score compared to VSE isolates (3 [IQR: 2–4] vs. 2 [IQR: 2–3]; *p* = 0.028, Mann–Whitney U test). The *esp* gene was more frequently detected among VRE isolates, while pheromone-associated genes displayed no clear group-specific pattern. **Conclusions**: Colonizing *E. faecalis* isolates carried a heterogeneous repertoire of the selected virulence-associated genes, with a higher burden of these genes among VRE isolates. These findings contribute additional data from Eastern Europe on the molecular characteristics of colonizing *E. faecalis* populations and support further investigation of their epidemiological relevance in healthcare settings.

## 1. Introduction

Antimicrobial resistance (AMR) represents a major global health concern, significantly limiting therapeutic options and increasing morbidity and mortality associated with bacterial infections [1]. Among Gram-positive pathogens, *Enterococcus faecalis* has emerged as one of the most relevant opportunistic microorganisms, frequently implicated in healthcare-associated infections such as bloodstream infections, endocarditis, urinary tract infections, and intra-abdominal infections [2,3,4]. Its intrinsic antimicrobial resistance to most beta lactams and aminoglycosides, ability to persist on abiotic surfaces, and remarkable genomic plasticity contribute to its successful adaptation to the hospital environment [3,5,6,7].

Vancomycin-resistant enterococci (VRE) are of particular clinical concern, as glycopeptides have long been considered last-resort antibiotics for the treatment of infections caused by multidrug-resistant Gram-positive bacteria [6,8]. The emergence and dissemination of VRE strains are primarily mediated by the acquisition of *van* gene clusters, most notably *vanA* and *vanB*, which confer high-level resistance to glycopeptides [9,10]. They are often located on transferable genetic elements, facilitating horizontal gene transfer and rapid clonal expansion in healthcare settings. Importantly, intestinal gastrointestinal carriage with VRE represents a critical epidemiological reservoir, preceding invasive infections and playing a central role in nosocomial transmission dynamics [11,12].

In *Enterococcus faecalis*, *quorum sensing* and pheromone-responsive systems contribute to the regulation of conjugative plasmid transfer and have been associated with virulence-related traits, including adhesion and biofilm formation [13,14,15]. Among the genes involved in these systems, *asa* encodes aggregation substance, *esp* encodes the enterococcal surface protein, *ccf* and *cpd* encode pheromone peptides involved in plasmid transfer, and *eep* encodes a membrane metalloprotease required for pheromone processing [16,17].

Although virulence factors in *Enterococcus* genus have been investigated in various clinical contexts, most available data derive from infection-associated isolates, with comparatively limited attention given to colonizing strains. This represents a significant gap, as gastrointestinal carriage constitutes the primary reservoir for transmission and subsequent infection. Moreover, existing studies report heterogeneous results regarding the prevalence of genes such as *esp* or *asa*, and their association with vancomycin resistance remains controversial, with some reports suggesting enrichment in hospital-adapted, resistant lineages, while others fail to demonstrate a consistent correlation [18,19].

Data regarding the distribution of pheromone-related genes (*ccf*, *cpd*) and regulatory elements, such as *eep*, in colonizing *E. faecalis* isolates are even more scarce, particularly in the context of VRE. Although virulence-associated genes in *E. faecalis* have been extensively investigated, molecular epidemiological evidence from Eastern Europe remains limited, with most studies relying on relatively small isolate collections and assessing only conventional virulence determinants [4,20,21]. Data regarding pheromone-response genes are particularly scarce, and no comprehensive molecular epidemiological studies are currently available from Romania. This lack of regional data limits our understanding of the virulome of colonizing *Enterococcus faecalis* isolates and its relationship with antimicrobial resistance [15,16,22]. In this context, molecular surveillance approaches targeting virulence-associated genes in colonizing isolates may provide valuable insights into the epidemiological potential of circulating strains and their capacity for persistence and dissemination within healthcare environments. We hypothesized that vancomycin-resistant *E. faecalis* isolates possess a higher prevalence of virulence-associated genes than vancomycin-susceptible isolates, potentially reflecting adaptation to hospital selective pressures [15,16,22].

These five targets were selected because they represent complementary aspects of *E. faecalis* pathogenicity, including adhesion (*esp*), aggregation and conjugative plasmid transfer (*asa*), pheromone signaling (*ccf* and *cpd*), and pheromone processing (*eep*), thereby providing a focused molecular overview of virulence-associated and pheromone-responsive determinants in colonizing isolates.

## 2. Materials and Methods

### 2.1. Study Design, Bacterial Isolation and Species Identification

This prospective laboratory-based observational study was conducted between 1st of January and 30th of June 2025 at the Microbiology Laboratory of the Cluj County Emergency Clinical Hospital, Cluj-Napoca, Romania. No clinical specimens were collected specifically for research purposes. Instead, the study included *Enterococcus faecalis* isolates recovered during routine hospital surveillance for vancomycin-resistant enterococci (VRE), performed as part of the hospital’s infection prevention and control program. Eligible isolates met the following inclusion criteria:

- recovery from a rectal screening swab collected during the study period as part of routine VRE surveillance;
- identification as *E. faecalis* by MALDI-TOF MS; and
- inclusion of only the first isolate recovered from each patient during the study period. Duplicate isolates obtained from repeated screening samples from the same patient were excluded.

Rectal swabs were processed according to the routine laboratory workflow. Samples were inoculated onto chromID VRE agar (bioMérieux, Marcy-l’Étoile, France) and incubated aerobically at 35–37 °C for 24–48 h according to the manufacturer’s recommendations. Colonies displaying morphology and chromogenic characteristics consistent with enterococci on chromID VRE agar were selected for further analysis.

Species identification was performed using matrix-assisted laser desorption ionization time-of-flight mass spectrometry (MALDI-TOF MS) with the Bruker Biotyper Sirius system (Bruker Daltonics, Bremen, Germany), according to the manufacturer’s instructions. Identification was accepted for log (score) values ≥ 2.0, corresponding to secure species-level identification. All included isolates subsequently underwent antimicrobial susceptibility testing according to EUCAST recommendations and PCR detection of the selected virulence-associated genes. No patient-identifiable or clinical data were collected. Based on antimicrobial susceptibility testing results, isolates were categorized as vancomycin-resistant (VRE) or vancomycin-susceptible (VSE).

### 2.2. Antimicrobial Susceptibility Testing

Antimicrobial susceptibility testing (AST) was performed by broth microdilution using Sensititre™ Gram Positive EUENCF plates (Thermo Fisher Scientific, USA), according to the manufacturer’s instructions. The panel includes antimicrobial agents representative of the major classes used for susceptibility testing of enterococci, including β-lactams (ampicillin and amoxicillin), glycopeptides (vancomycin and teicoplanin), oxazolidinones (linezolid), glycylcyclines (tigecycline), fluoroquinolones (ciprofloxacin, levofloxacin and norfloxacin), carbapenems (imipenem), trimethoprim, nitrofurantoin, quinopristin/dalfopristin, and high-level aminoglycoside resistance markers (gentamicin and streptomycin).

Bacterial suspensions equivalent to 0.5 McFarland were prepared in sterile water and diluted in Sensititre Mueller–Hinton broth (Thermo Fisher Scientific, USA). Plates were inoculated using the Sensititre AIM™ system (50 μL per well) and incubated at 35 °C for 18–24 h using the ARIS™ system. Quality control was performed using *Enterococcus faecalis* ATCC 29212 in accordance with the manufacturer’s recommendations and EUCAST quality-control guidelines.

Minimum inhibitory concentrations (MICs) were interpreted according to the European Committee on Antimicrobial Susceptibility Testing (EUCAST) clinical breakpoints (version 16.0, 2026). Isolates with a vancomycin MIC > 4 mg/L were classified as vancomycin-resistant (VRE), whereas isolates with a MIC ≤ 4 mg/L were classified as vancomycin-susceptible (VSE). Since the primary objective of this study was to compare virulence-associated genes between VRE and VSE isolates, vancomycin susceptibility was used solely to define the study groups, while susceptibility results for the remaining antimicrobial agents were not included in the present analysis.

### 2.3. Molecular Detection of the Virulence Genes

The presence of virulence-associated genes (*asa*, *esp*, *eep*, *ccf*, and *cpd*) was investigated by polymerase chain reaction (PCR). PCR amplification was performed directly from bacterial colony suspensions without prior genomic DNA extraction, following a previously published protocols adapted for the present study [23,24]. Briefly, several well-isolated colonies were suspended in sterile distilled water to a turbidity equivalent to 0.5 McFarland, and 2 μL of the resulting bacterial suspension was used directly as the PCR template.

Each PCR reaction was performed in a final volume of 25 μL containing 12.5 μL DreamTaq Green PCR Master Mix (2×) (Thermo Fisher Scientific, Waltham, MA, USA), 10.25 μL nuclease-free water (Lonza, Basel, Switzerland), 25 pmol of each primer (Eurogentec, Liège, Belgium), and 2 μL of bacterial suspension. A reaction containing sterile nuclease-free water instead of bacterial suspension was included as a negative control. Amplification was carried out using a Mastercycler Nexus thermocycler (Eppendorf, Hamburg, Germany). The PCR protocol consisted of an initial denaturation step at 94 °C for 4 min, followed by 35 amplification cycles of denaturation at 94 °C for 30 s, annealing for 45 s at the primer-specific temperature (Table 1), and extension at 72 °C for 45 s, with a final extension step at 72 °C for 8 min.

PCR products were separated by electrophoresis on 1.5% (*w*/*v*) agarose gel (Cleaver Scientific, Rugby, Warwickshire, UK) prepared in 1× TAE buffer (Lonza, Basel, Switzerland), stained with ethidium bromide, and visualized under ultraviolet illumination.

A virulence score was calculated for each isolate based on the number of detected virulence genes (*asa*, *esp*, *eep*, *ccf*, and *cpd*), with one point assigned for each gene present, resulting in a score ranging from 0 to 5.

### 2.4. Statistical Analysis

Statistical analysis was performed using IBM SPSS Version 31.0 (IBM Corp., 2025). Statistics. Categorical variables (presence or absence of virulence-associated genes) were compared between VRE and VSE isolates using Fisher’s exact test. Continuous variables (cumulative virulence gene score) were expressed as median and interquartile range (IQR) and compared using the Mann–Whitney U test. Owing to the limited number of VRE isolates, multivariable logistic regression analysis was not performed, as the sample size was insufficient to support a robust multivariable model. Statistical significance was defined as *p* < 0.05. Because five independent comparisons were performed, a Bonferroni-adjusted significance threshold of *p* < 0.01 was also considered.

### 2.5. Ethical Considerations

This study was conducted exclusively on anonymized bacterial isolates recovered during routine diagnostic procedures. No patient-identifiable information was collected, no additional clinical specimens were obtained for research purposes, and no patient intervention was performed. According to institutional regulations, formal ethics committee approval was not required for studies conducted exclusively on anonymized bacterial isolates.

## 3. Results

A total of 44 *Enterococcus faecalis* isolates obtained from rectal screening samples were included in the analysis. Based on antimicrobial susceptibility testing, 14 isolates (31.8%) were classified as vancomycin-resistant (VRE), while 30 isolates (68.2%) were vancomycin-susceptible (VSE).

**Figure 1:**
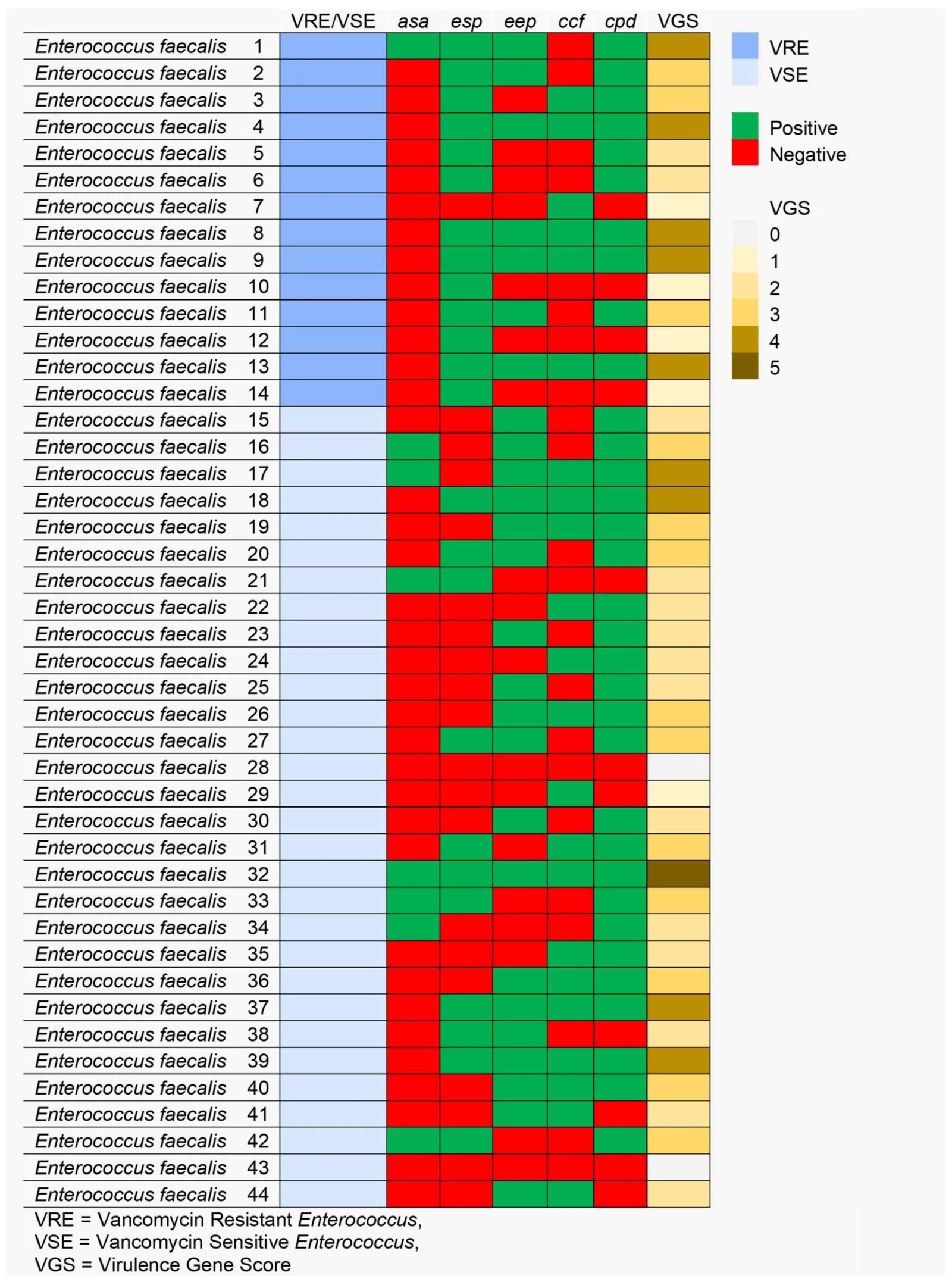
Heatmap illustrating the distribution of virulence-associated genes (*asa*, *esp*, *eep*, *ccf*, and *cpd*) among Enterococcus faecalis isolates.

To provide an overview of the distribution and co-occurrence patterns of these virulence determinants, a heatmap was constructed (Figure 1), allowing visualization of gene profiles at the individual isolate level and their relationship with vancomycin resistance phenotype.

The prevalence of virulence-associated genes among *E. faecalis* isolates is presented in Table 2. Among the investigated determinants, *esp* was the most prevalent gene overall and demonstrated a significantly higher frequency in VRE isolates compared to VSE (13/14, 92.9% vs. 11/30, 36.7%; *p* < 0.01). In contrast, no statistically significant differences were observed between VRE and VSE isolates for the other investigated genes, including *asa* (7/30 vs. 1/14; *p* = 0.38), *eep* (19/30 vs. 7/14; *p* = 0.52), *ccf* (16/30 vs. 6/14; *p* = 0.75), and *cpd* (23/30 vs. 10/14; *p* = 1). Overall, *cpd* and *eep* were among the most frequently detected genes across both groups, whereas *asa* showed the lowest prevalence.

A wide diversity of virulence gene combinations was observed among the analyzed isolates (Table 3). In the VRE group, the most common genotype corresponded to the combination *esp-eep-ccf-cpd* (4/14, 28.6%), followed by *esp* alone (3/14, 21.4%) and *esp, eep, cpd* (2/14, 14.3%). Moreover, most VRE isolates harbored multiple virulence determinants, with only a single isolate carrying a single gene (*ccf*). In the VSE group, a broader distribution of gene combinations was identified, with the most frequent profile being *eep-cpd* (5/30, 16.7%), followed by *eep-ccf-cpd* (4/30, 13.3%). A small proportion of isolates (2/30, 6.7%) did not carry any of the investigated virulence genes. Overall, VRE isolates tended to exhibit more complex virulence gene profiles, whereas VSE isolates showed greater heterogeneity, including combinations with fewer or no detected genes.

**Figure 2:**
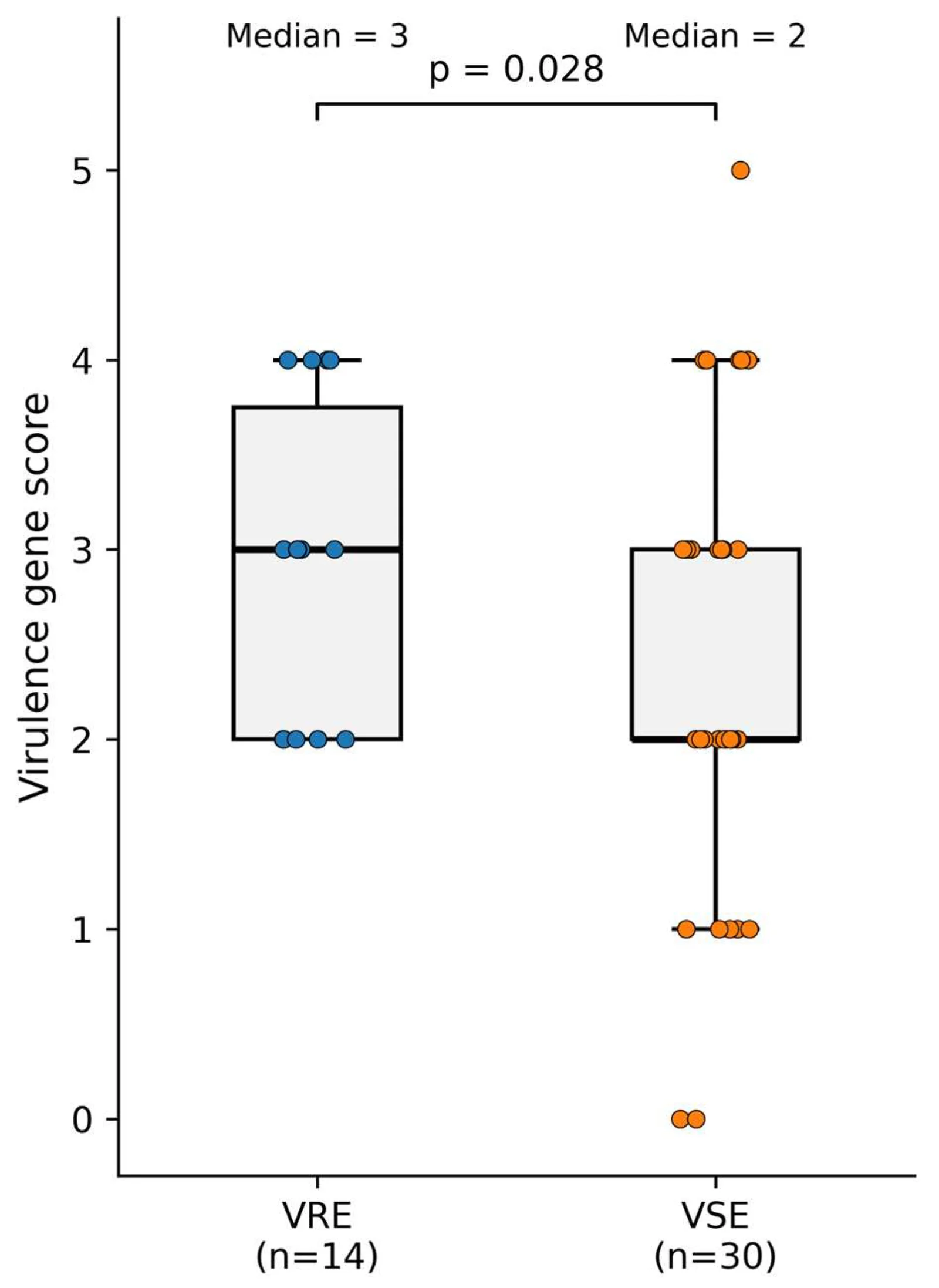
Comparison of cumulative virulence gene scores in colonizing vancomycin-resistant (VRE) and vancomycin-susceptible (VSE) Enterococcus faecalis isolates. The cumulative virulence gene score (VGS) was calculated as the sum of five virulence-associated genes (*asa*, *esp*, *eep*, *ccf*, and *cpd*), resulting in a score ranging from 0 to 5. Boxes represent the interquartile range (IQR), the horizontal line indicates the median, whiskers extend to 1.5 × IQR, and each dot represents an individual isolate. Statistical significance was assessed using the Mann–Whitney U test.

VRE isolates exhibited a modest but statistically significant increase in the cumulative virulence gene score compared with VSE isolates (median 3 [IQR 2–4] vs. 2 [IQR 2–3], Mann–Whitney U test, *p* = 0.028). Given the limited sample size and overlapping interquartile ranges, this finding should be interpreted with caution (Figure 2).

## 4. Discussion

### 4.1. Main Findings

The present study investigated the distribution of selected virulence-associated genes among colonizing *Enterococcus faecalis* isolates and their relationship with vancomycin resistance. Overall, a heterogeneous distribution of virulence determinants was observed across the study population. Notably, VRE isolates exhibited a higher cumulative virulence gene score compared to VSE isolates, with a statistically significant difference between the two groups. In addition, certain genes, particularly *esp*, appeared more frequently among VRE isolates. In contrast, other targets, including pheromone-associated genes, were variably distributed without a clear pattern of exclusivity. Together, these findings suggest that colonizing *E. faecalis* isolates harbor a diverse repertoire of virulence-associated genes, with a tendency toward a higher cumulative virulence gene burden among vancomycin-resistant isolates.

The distribution of virulence-associated genes observed in this study highlights the complex pathogenic potential of colonizing *E. faecalis* isolates. Traditionally regarded as commensals of the gastrointestinal tract, enterococci have increasingly been recognized as opportunistic pathogens capable of causing a wide range of infections, particularly in hospitalized patients [25]. In this context, the presence of virulence determinants in colonizing isolates is of particular relevance, as these isolates may serve as a reservoir for subsequent infection or horizontal gene transfer.

Among the investigated genes, *asa* and *esp* have been extensively associated with adhesion, biofilm formation, and persistence within the host [26,27,28]. The detection of these genes in a substantial proportion of isolates aligns with previous reports indicating that colonizing strains may already possess key attributes required for host interaction and environmental survival [25,26,27,28]. Moreover, *esp* has been frequently linked to hospital-adapted lineages and has been proposed as a marker of strains with enhanced epidemic potential [12,28,29,30].

Furthermore, the identification of genes associated with pheromone-responsive systems, including *ccf*, *cpd*, and the regulatory gene *eep*, suggests the genetic potential for pheromone-mediated interbacterial communication among colonizing isolates [16,31].

Importantly, the observed gene distribution does not appear to be restricted to isolates derived from clinical infections, reinforcing the idea that the boundary between colonization and pathogenicity in *E. faecalis* is fluid. Rather than representing a biologically inert population, colonizing strains may harbor virulence-associated genetic determinants that have previously been implicated in host interaction and persistence. However, the present study did not evaluate their phenotypic expression or their association with progression to invasive disease.

### 4.2. Association Between Vancomycin Resistance and Virulence

A central finding of this study is the association between vancomycin resistance and an increased burden of virulence-associated genes in colonizing *E. faecalis* isolates. Specifically, VRE isolates displayed a significantly higher cumulative virulence score compared to VSE isolates, suggesting that resistance and virulence traits may co-occur within the same bacterial population. In addition, the *esp* gene was more frequently detected among VRE isolates, further supporting a potential link between resistance and factors involved in host interaction and persistence. These results concur with existing data [29,32,33].

Although the mechanisms underlying this association cannot be directly elucidated in the absence of genomic data, several hypotheses may explain this observation. One plausible explanation is the co-selection of resistance and virulence determinants under hospital-related selective pressures, particularly in high-risk environments such as intensive care units [29,33]. In such settings, the extensive use of antimicrobials may favor the persistence and spread of strains that combine antimicrobial resistance with enhanced adaptive and colonization capabilities [25,28,34].

Another possible explanation is the expansion of hospital-adapted clonal lineages that inherently carry both resistance and virulence-associated genes. Previous studies have shown that certain high-risk enterococcal lineages are characterized by the accumulation of mobile genetic elements encoding both antimicrobial resistance and virulence factors, including *esp* [12,29,33]. In this context, the higher cumulative virulence gene burden observed among VRE isolates may reflect the genetic background of successful nosocomial clones rather than a direct functional relationship between antimicrobial resistance and the expression of virulence traits.

It is also important to consider the role of mobile genetic elements in facilitating the co-localization and dissemination of these traits [12]. Enterococci are well known for their ability to acquire and exchange genetic material via plasmids and transposons, many of which may carry multiple adaptive determinants. The concomitant presence of pheromone-responsive genes in the studied isolates further supports the potential for active horizontal gene transfer within this population [12,25,35].

However, the cross-sectional design of this study and the lack of whole-genome sequencing data preclude definitive conclusions regarding causality or genetic linkage. Therefore, the observed association should be interpreted as indicative of co-occurrence rather than a direct mechanistic relationship. Further studies integrating genomic and functional approaches are needed to clarify whether virulence and resistance determinants are physically linked or simply selected together under similar environmental pressures.

### 4.3. Comparison with Studies from Other Geographic Regions

The findings of the present study are broadly consistent with recent reports from different geographical regions, suggesting that the coexistence of virulence-associated genes and antimicrobial resistance is not restricted to European *Enterococcus faecalis* isolates. Nevertheless, differences in isolate origin, investigated virulence markers, antimicrobial resistance profiles, and study design should be considered when making direct comparisons [35,35,36,37].

In Africa, Georges et al. investigated clinical *Enterococcus* isolates collected in Kenya [28] and reported that 72.9% of *E. faecalis* isolates carried multiple virulence genes [36]. Furthermore, some genes were significantly associated with several antimicrobial resistance phenotypes, supporting the concept that virulence-associated and resistance determinants may frequently coexist within the same bacterial population [28,36].

Similar observations have been reported in Asia. An Indian molecular epidemiology study demonstrated a prevalence of 36.6% for *esp*, among multidrug-resistant *E. faecalis* isolates and showed that highly successful hospital-associated clonal complexes (CC2 and CC87) accumulated multiple virulence determinants together with antimicrobial resistance traits [38].

Comparable findings have also been described in Egypt, where *E. faecalis* urinary isolates frequently carried several virulence-associated genes simultaneously, although no single virulence profile was uniquely associated with a specific clinical presentation, highlighting the complexity of the relationship between genotype and phenotype [39].

Evidence from South America further supports the widespread distribution of these virulence determinants. A Brazilian study evaluating clinical *E. faecalis* isolates demonstrated frequent detection and transcriptional activity of *esp* under different antimicrobial conditions, reinforcing its biological relevance while also illustrating that gene presence alone cannot be equated with constitutive expression or phenotypic virulence [35].

Collectively, these studies indicate that the coexistence of antimicrobial resistance and virulence-associated genes is a globally observed phenomenon rather than one restricted to European isolates. Nevertheless, important methodological differences—including isolate source, antimicrobial resistance profiles, investigated virulence markers, and molecular approaches—limit direct comparison between studies and should be considered when interpreting these findings [35,36,37,38,39].

### 4.4. Quorum Sensing and Pheromone-Responsive Systems

In addition to classical virulence determinants, this study investigated the distribution of genes associated with pheromone-responsive systems, namely *ccf*, *cpd*, and *eep*. These genes are involved in pheromone signaling and processing and have previously been associated with conjugative plasmid transfer and the regulation of selected virulence-related traits in *E. faecalis* [16,40].

The detection of these genes in a substantial proportion of colonizing isolates indicates the presence of genetic determinants associated with pheromone-responsive systems during asymptomatic carriage [41]. However, because the present study was based exclusively on PCR detection, no conclusions can be drawn regarding gene expression, signaling activity, or their functional contribution to plasmid transfer, virulence, or persistence.

Although these findings suggest that colonizing isolates harbor genes associated with pheromone-responsive systems, further transcriptomic and functional studies are required to determine whether these genetic determinants are actively expressed and contribute to the biology of colonizing *E. faecalis* strains.

### 4.5. Clinical and Epidemiological Implications

The findings of this study have several potential clinical and epidemiological implications, particularly in the context of hospital-associated colonization. The identification of a substantial virulence gene repertoire among colonizing *E. faecalis* isolates suggests that these strains should not be regarded as biologically inert. Instead, they may represent a reservoir of genetically equipped organisms with the capacity to transition from asymptomatic carriage to invasive infection, especially in vulnerable patients. Also, the presence of pheromone-responsive and quorum sensing-related genes raises additional concerns regarding the potential for horizontal gene transfer within the intestinal microbiota. The gastrointestinal tract provides a favorable environment for bacterial interaction and genetic exchange, which may contribute to the dissemination of resistance determinants across enterococcal populations and potentially to other Gram-positive organisms [7,21,42,43].

From an infection control perspective, these findings support the continued importance of active surveillance strategies for VRE gastrointestinal carriage. However, they also suggest that a more nuanced understanding of colonizing strains—beyond resistance profiles alone—may be warranted. The integration of virulence-associated markers into epidemiological studies could provide additional insights into the molecular epidemiology of colonizing *E. faecalis* isolates and the dynamics of nosocomial dissemination [7].

Overall, this study underscores the need to consider colonizing *E. faecalis* isolates as part of a dynamic and clinically relevant reservoir, with implications for both infection prevention and the broader ecology of antimicrobial resistance in healthcare settings.

### 4.6. Methodological Considerations

An important methodological consideration relates to the initial recovery of isolates on chromogenic VRE screening media. Although all isolates were recovered from selective VRE agar, confirmatory antimicrobial susceptibility testing demonstrated that not all fulfilled the criteria for vancomycin resistance. This finding reflects the known limitations of chromogenic screening media, which are designed to maximize sensitivity at the expense of specificity and may occasionally permit the growth of vancomycin-susceptible enterococci [22,41,42]. Consequently, growth on selective media should always be confirmed by standardized susceptibility testing. Nevertheless, inclusion of these isolates provides a realistic representation of the enterococcal population recovered during routine surveillance screening [22,41,42].

Other methodological aspects should also be considered when interpreting the findings of this study. First, the selection of virulence-associated genes was based on their previously reported involvement in adhesion, colonization, biofilm formation, and interbacterial communication [16]. This targeted approach allowed for a focused evaluation of key functional pathways relevant to both virulence and horizontal gene transfer.

It is important to mention that the presence of selected virulence-associated genes was investigated by PCR, without assessing their expression or functional activity. Therefore, the detected genes should be interpreted as indicators of genetic potential rather than phenotypic virulence.

In addition, biofilm formation was not evaluated phenotypically, precluding correlation between the identified virulence-associated genes and biofilm-producing capacity. Third, vancomycin resistance was established by standardized antimicrobial susceptibility testing, whereas molecular confirmation of vancomycin resistance genes (e.g., *vanA* or *vanB*) was not performed.

Also, the calculation of a cumulative virulence gene score enabled a simplified comparison between groups, facilitating statistical analysis and interpretation. While this approach is useful for capturing the overall burden of virulence-associated determinants, it does not account for differences in the relative contribution or biological impact of individual genes. Finally, the relatively small number of VRE isolates from a single center may limit the generalizability of our findings.

### 4.7. Limitations

This study has several limitations that should be acknowledged. First, the relatively small number of isolates, particularly in the VRE group, may limit the generalizability of the findings and the statistical power of subgroup analyses [43]. Second, all isolates were derived from colonization screening samples, without associated clinical data, precluding any direct correlation between virulence gene profiles and clinical outcomes.

Another important limitation is the absence of whole-genome sequencing data. As a result, it was not possible to determine the genetic context of the detected genes, including their localization on chromosomes or mobile genetic elements, nor to assess clonal relationships between isolates. This also limits the ability to explore potential linkage between antimicrobial resistance and virulence determinants at the genomic level.

Furthermore, the study did not include functional assays, such as biofilm formation or gene expression analyses, which could provide additional insight into the phenotypic relevance of the detected genes. Therefore, the findings should be interpreted as indicative of genetic potential rather than confirmed virulence behavior.

Finally, the cross-sectional design captures a single time point and does not allow assessment of the temporal dynamics of colonization, persistence, or progression to infection. Longitudinal studies would be required to better understand the clinical significance of these colonizing strains.

### 4.8. Future Directions

Future research should focus on refining the understanding of how colonizing *Enterococcus faecalis* populations contribute to the broader ecology of antimicrobial resistance and hospital adaptation. Rather than assessing individual determinants in isolation, integrative approaches that capture the interaction between bacterial communities, host factors, and environmental pressures may provide a more comprehensive perspective on colonization dynamics.

In this context, exploring the role of the intestinal microbiota as a reservoir and interaction network for enterococci could offer valuable insights into the mechanisms that facilitate persistence and dissemination. Investigating how selective pressures shape the composition and functional potential of colonizing populations may help clarify the conditions under which these strains acquire increased clinical relevance.

Another promising direction involves the identification of predictive markers associated with stable gastrointestinal carriage versus transition toward infection. Moving beyond descriptive profiling, future studies could aim to define composite risk signatures that integrate microbiological and ecological parameters, potentially supporting more targeted surveillance and infection prevention strategies.

Finally, expanding research efforts toward translational applications or strategies to limit colonization and transmission, may contribute to reducing the burden of enterococcal infections in healthcare settings.

## 5. Conclusions

In conclusion, this study demonstrates that colonizing *Enterococcus faecalis* isolates harbor a diverse repertoire of virulence-associated genes, underscoring their potential clinical relevance beyond asymptomatic carriage. The observed association between vancomycin resistance and a higher virulence gene burden suggests a co-occurrence of antimicrobial resistance and virulence-associated genetic determinants among the studied isolates. In addition, the frequent detection of *quorum sensing* genes indicates that colonizing isolates possess the genetic determinants associated with these systems, although their functional activity was not evaluated in the present study.

Importantly, this study provides novel data on the distribution of virulence-associated genes among colonizing *E. faecalis* isolates in an Eastern European setting, a region for which such data remain relatively limited. By focusing on isolates recovered through routine VRE screening, these findings reflect real-world laboratory and epidemiological conditions and provide additional insight into the molecular characteristics of colonizing enterococcal populations encountered in clinical practice.

Overall, these findings support the view that colonizing *E. faecalis* isolates constitute an important reservoir of virulence-associated genetic determinants. Future studies integrating genomic, transcriptomic, and phenotypic approaches are warranted to clarify the functional significance of these genes and their contribution to colonization, persistence, and the dissemination of antimicrobial resistance in healthcare settings.

## Figures and Tables

**Table 1:** PCR primers users and annealing temperatures for gene amplifications.

No.	Gene Name	Primer Direction	Primer Sequence (5′-3′)	Annealing Temperature (°C)	Amplicon Size (bp)
1	*esp*	Forward	5′ CTTTGATTCTTGGTTGTCGGATAC-3′	63	475
		Reverse	5′ TCCAACTACCACGGTTTGTTTATC-3′		
2	*asa*	Forward	5′ GCACGCTATTACGAACTATGA-3′	60	357
		Reverse	5′ TAAGAAAGAACATCACCACGA-3′		
3	*eep*	Forward	5′ GAGCGGGTATTTTAGTTCGT-3′	60	937
		Reverse	5′ TACTCCAGCATTGGATGCT-3′		
4	*ccf*	Forward	5′ GGGAATTGAGTAGTGAAGAAG-3′	59	543
		Reverse	5′ AGCCGCTAAAATCGGTAAAAT-3′		
5	*cpd*	Forward	5′ TGGTGGGTTATTTTTCAATTC-3′	58	782
		Reverse	5′ TACGGCTCTGGCTTACTA-3′		

**Table 2:** Distribution of virulence-associated genes (*asa*, *esp*, *eep*, *ccf*, and *cpd*) among *Enterococcus faecalis* isolates organized by vancomycin resistance phenotype (VRE vs. VSE). * *p* < 0.05.

No.	Gene	VSE (N = 30)	VRE (N = 14)	OR (95% CI) VSE vs. VRE	*p*
1.	*asa*	7	1	3.96 (0.44–35.81)	0.38
2.	*esp*	11	13	0.04 (0.005–0.39)	<0.01 *
3.	*eep*	19	7	1.73 (0.48–6.24)	0.52
4.	*ccf*	16	6	1.52 (0.42–5.47)	0.75
5.	*cpd*	23	10	1.31 (0.31–5.52)	1

**Table 3:** Most frequent virulence genotype combinations identified among VRE and VSE Enterococcus faecalis isolates. Only genotype combinations observed in at least two isolates are presented. Rare genotype combinations (identified in a single isolate) are illustrated in the heatmap (Figure 1). Statistical comparisons between VRE and VSE isolates were performed using Fisher’s exact test.

Genotype Combination	VSE (n = 30) n (%)	VRE (n = 14) n (%)	*p*-Value †
*esp*, *eep*, *ccf*, *cpd*	3 (10.0)	4 (28.6)	0.184
*eep*, *ccf*, *cpd*	4 (13.3)	0 (0.0)	0.290
*esp*, *eep*, *cpd*	2 (6.7)	2 (14.3)	0.581
*asa*, *esp*, *cpd*	2 (6.7)	0 (0.0)	1.000
*eep*, *cpd*	5 (16.7)	0 (0.0)	0.161
*ccf*, *cpd*	3 (10.0)	0 (0.0)	0.540
None	2 (6.7)	0 (0.0)	1.000

† Fisher’s exact test (two-tailed).

## Data Availability

The original contributions presented in this study are included in the article. Further inquiries can be directed to the corresponding author(s).
